# Type II collagen and glycosaminoglycan expression induction in primary human chondrocyte by TGF-β1

**DOI:** 10.1186/s12891-015-0599-x

**Published:** 2015-06-10

**Authors:** Hyun Joo Yoon, Suk Bum Kim, Dhara Somaiya, Moon Jong Noh, Kyoung-Baek Choi, Chae-Lyul Lim, Hyeon-Youl Lee, Yeon-Ju Lee, Youngsuk Yi, Kwan Hee Lee

**Affiliations:** TissueGene Inc., 9605 Medical Center Dr. Suite 200, Rockville, MD 20850 USA; Department of Rehabilitation and Personal training, Konyang University, 158, Gwanjeodong-ro, Daejeon, Seo-gu Korea; Kolon Life Science, 13 Kolon-ro, Gwacheon-si, Gyeonggi-do Korea

## Abstract

**Background:**

A localized non-surgical delivery of allogeneic human chondrocytes (hChonJ) with irradiated genetically modified chondrocytes (hChonJb#7) expressing transforming growth factor-β1 (TGF-β1) showed efficacy in regenerating cartilage tissue in our pre-clinical studies and human Phase I and II clinical trials. These previous observations led us to investigate the molecular mechanisms of the cartilage regeneration.

**Methods:**

Genetically modified TGF-β1preprotein was evaluated by monitoring cell proliferation inhibition activity. The effect of modified TGF-β1 on chondrocytes was evaluated based on the type II collagen mRNA levels and the amount of glycosaminoclycan (GAG) formed around chondrocytes, which are indicative markers of redifferentiated chondrocytes. Among the cartilage matrix components produced by hChonJb#7 cells, type II collagen and proteoglycan, in addition to TGF-β1, were also tested to see if they could induce hChonJ redifferentiation. The ability of chondrocytes to attach to artificially induced defects in rabbit cartilage was tested using fluorescent markers.

**Results:**

Throughout these experiments, the TGF-β1 produced from hChonJb#7 was shown to be equally as active as the recombinant human TGF-β1. Type II collagen and GAG production were induced in hChonJ cells by TGF-β1 secreted from the irradiated hChonJb#7 cells when the cells were co-cultured in micro-masses. Both hChonJ and hChonJb#7 cells could attach efficiently to the defect area in the rabbit cartilage.

**Conclusions:**

This study suggests that the mixture (TG-C) of allogeneic human chondrocytes (hChonJ) and irradiated genetically modified human chondrocytes expressing TGF-β1 (hChonJb#7) attach to the damaged cartilage area to produce type II collagen-GAG matrices by providing a continuous supply of active TGF-β1.

## Background

Deterioration of cartilage leads to osteoarthritis, through which articular chondrocytes become deformed, or fibrillated, losing their cartilage forming function. Additionally, once damaged or injured, cartilage has limited capability for self-healing due to its avascular characteristics [1]. Transplantation of autologous or allogeneic chondrocytes, or mesenchymal stem cells are promising treatments for cartilage damage [2, 3]. However, due to limited supplies of autologous or allogeneic chondrocytes and technical difficulties associated with differentiating stem cells to chondrocytes, a reliable method for repairing cartilage damage remains undeveloped [4, 5].

Although small population of other cell type such as mesenchymal progenitor cells have been observed, mature chondrocytes are the major cell type which exists in cartilage and are the ultimate cell type for treating cartilage damage [6, 7]. Mature chondrocytes have been shown to exhibit a continual loss of their chondrocyte characteristics during extended monolayer expansion [8]. Expression of type II collagen, one of the prominent components of cartilage, decreases during the growth of chondrocytes in two dimensional culture vessels, while type I collagen increases significantly [9]. The original round or polygonal shape of chondrocytes also changes to a spindled, fibroblast-like shape which is one of the characteristics of a “de-differentiated” state. Chondrocytes return to a “re-differentiated” state in three dimensional cultures in matrices such as agarose [10]. Because the response of chondrocytes is sensitive to subtle differences in surface roughness and surface chemistry, a natural environment change such as surface damage is also assumed to affect the characteristics of chondrocytes [11]. This chondrocyte-matrix interaction plays a contributing role in maintaining and repairing articular cartilage [12].

The substance of the matrix is considered important for cartilage regeneration. Cartilage defects treated with type I collagen matrices showed fibro-cartilage tissue generation while defects treated with type II collagen matrices produced hyaline cartilage tissue [13]. In addition to the matrix, bioactive factors added to chondrocytes or mesenchymal stem cells have been used to maintain the integrity of chondrocytes [14, 15]. A combination of basic fibroblast growth factor (bFGF) and type I/II collagen matrix showed increased GAG expression compared with the addition of each component singly [16]. GAG synthesis was also stimulated by TGF-β1, which is a known induction factor for the differentiation of stem cells to cartilage [17, 18].

We have reported that the re-differentiated status of chondrocytes and induced cartilage regeneration could be maintained by providing a continuous supply of TGF-β1 [19–21]. Additionally, we have found that providing chondrocytes with TGF-β1 producing cells helps cartilage regeneration [22]. Our pre-clinical studies showed the production of hyaline cartilage using a mixture (TG-C) of human chondrocytes (hChonJ) and irradiated TGF-β1 producing chondrocytes (hChonJb#7) [23]. To elucidate the mechanism of cartilage regeneration, the effects of TGF-β1produced from irradiated hChonJb#7 in TG-C on the de-differentiated chondrocytes were investigated. Additionally, to evaluate the adherence of TG-C when injected directly without a scaffold, an assessment of TG-C binding to the damaged cartilage surface was performed. This study suggests the mechanism of new cartilage formation by TG-C, in which chondrocytes bound to the damaged cartilage accumulate ECM including type II collagen and GAG through continuous induction of TGF-β1 produced from co-injected hChonJb#7cells. This, therefore, provides a favourable environment for chondrocyte re-differentiation eventually resulting in cartilage regeneration.

## Methods

### Cell culture

Human cartilage samples were obtained at the time of surgery for excision of a polydactyl finger. The patient was one year old. Cartilage tissues were obtained from the rudimentary excised finger, which was donated for further experiments by parents with written consent. The study was performed in compliance with the ethical guidelines of the Helsinki Declaration, and was approved by the institutional review board at the Inha University Medical School and Hospital.

Excised cartilage tissues were rinsed with sterile phosphate buffered saline (PBS) and treated with collagenase (from Clostridium histolyticum; GIBCO-BRL/Invitrogen, Rockville, MD) in Ham’s F12 medium containing 10 % fetal bovine serum (FBS). After collagenase treatment, the cartilage tissues were strained to collect chondrocyte cells. Chondrocytes were washed with PBS and cultured in Ham’s F12 medium containing 10 % FBS [19].

The primary chondrocytes (hChonJ) and the genetically modified clone, hChonJb#7 were expanded with Dulbecco’s modified Eagle’s medium (DMEM) containing 10 % FBS and 1 % L-Glutamine in a 37 °C, 5 % CO_2_ atmosphere.

For micro-mass culture, 3 × 10^5^ hChonJ cells (passage 8) or a mixture of 3 × 10^5^ hChonJ cells and 1 × 10^5^ irradiated hChonJb#7 cells (passage 18) in 20 μl of chondrogenic medium were seeded in each well of type I collagen coated 24 well plates. After cells were allowed to adhere at 37 °C for 1.5 h, 1 ml of chondrogenic medium was carefully added to each well. When required, recombinant human TGF-β1 (25 ng/ mass if not designated, eBioscience, San Diego, CA, USA), purified human type II collagen (25 ng/ mass, EMD Millipore, Temecula, CA, USA), or Proteoglycans (25 ng/ mass ProteaImmun, Berlin, Germany) was added into the seeding cell mixture. Culture medium was changed every 3–4 days with supplemented chondrogenic medium. For chondrogenic medium preparation, 0.1 mM dexamethasone, 0.17 mM ascorbic acid, 0.35 mM proline, 1 mM Sodium pyruvate, and Insulin-transferrin-salenous acid (1×) were supplemented into serum free high glucose DMEM. Micro-masses were harvested on day 7 to use for tests.

For culture and expansion of COS-1 monkey kidney cells (ATCC CRL-1650) and Hep G2 Hepatocellular Carcinoma (ATCC HB-8065), ATCC guidance was followed using 10 % FBS containing DMEM and Eagle’s Minimum Essential Medium (EMEM) respectively.

### Construct of wild-type TGF-β1 and mutants

To investigate of the biological activity of TGF-β1 produced from hChonJb#7, we made four constructs as follows: wild-type TGF-β1, TGF-β1^S223/225^ containing a serine at position 223 and 225 of the TGF-β1 precursor in place of the wild-type cysteine residue, TGF-β1^A84/W255^ containing a alanine and tryptophan at position 84 and 255 of the TGF-β1 precursor in place of the wild-type threonine and arginine residue, respectively, and TGF-β1^hChonJb#7^ containing all of four residue mutations, which is produced from hChonJb#7. To construct vector containing wild-type TGF-β1 and mutants, the coding region of wild-type TGF-β1 and TGF-β1^hChonJb#7^ was amplified by PCR using cDNA synthesized by the RNA of normal chondrocyte and pKEB1 plasmid which was used to manufacture hChonJb#7 cell line and then inserted into the Xho I and EcoR I sites of pMSCV retroviral vector (Clontech, CA, USA). Afterwards, TGF-β1^S223/225^ and TGF-β1^A84/W255^ were respectively constructed from wild-type TGF-β1 and TGF-β1^hChonJb#7^ constructs using a point-mutagenesis kit according to the manufacturer’s protocol (Agilent Technologies, CA, USA).

### Western blot analysis

To confirm the expression of TGF-β1 constructs, culture supernatant was mixed with reducing or non-reducing (with or without β-mercaptoethanol, respectively) Tris-Glycine sample buffer and separated by sodium dodecyl sulfate-polyacrylamide gel electrophoresis (SDS-PAGE). Electrotransferred nitrocellulose membrane was probed with anti-TGF-β1 antibody (Cell Signaling Technology, Inc., Beverly, MA, USA).

### TGF-β1 protein bioactivity assay

The four TGF-β1 constructs transfected Cos-1 cell culture medium was used for TGF-β1 source to monitor proliferation inhibition of Hep G2 hepatocellular carcinoma. The concentration of TGF-β1 in the Cos-1 culture medium was measured by Enzyme-linked Immunosorbent Assay (ELISA) after acid activation and a designated amount of TGF-β1, which was expected to be if activated, was applied into Hep G2 cell culture (seeded at a cell density of 5000 cells/well of 96 μ-well plate). After 1 week incubation, the levels of viable cells were measured using Cell Counting Kit-8 (CCK-8) assay (Dojindo Molecular Technologies, Inc., Rockville, MD, USA).

### *In vitro* cartilage formation

Culture-expanded hChonJ (passage 8) cells were thawed at 37 °C and washed in phosphate buffered saline (PBS) and then resuspended in a chondrogenic medium. Aliquots of 2.0 × 10^5^ cells, suspended in 1 mL chondrogenic medium supplemented with 25 ng/mL of recombinant human TGF-β1 (Peprotech, NJ, USA), were centrifuged at 450 × g for 5 min in 15 mL polypropylene conical tubes. Pelleted cells were incubated at 37 °C, 5 % CO_2_ atmosphere with loosened caps to permit gas exchange. The medium was changed every 3–4 days and pellets were harvested on days 21. Safranin-O staining and immunohistochemical staining of type II collagen were performed. This experiment was performed four times, making 3 pellets each time.

### Histological staining

At 21 days, pellets were embedded in optimum cutting temperature (OCT) compound and transferred the frozen pellet blocks to −20 °C of a Cryotome™ E cryostat (Thermo Scientific, MA, USA). Blocks of the pellets were cut into 3 μm thick slices using a cryostat and mounted on glass slides. The sections were stained with Safranin-O (S-O) for proteoglycan and immunohistochemistry for type II collagen. For S-O staining, sections were incubated in distilled water for 10–15 min and 0.1 % S-O solution was applied for 5 min, and then washed with distilled water. For immunohistochemistry, we used the Histostatin-Plus Kit (Invitrogen, CA, USA). After sections were incubated in distilled water for 10–15 min, washed with PBS for 10 min, and then, we performed according to the manufacturer’s protocol. The primary antibody against human type II collagen (EMD Millipore) was used and incubated at 4 °C overnight.

### Glycosaminoglycan (GAG) assay

The total soluble GAG of cultured micro-masses was measured using a Blyscan sulfated glycosaminoglycan assay kit (biocolor, Carrickfergus, County Antrim, UK). The micro-mass in each well was washed with PBS and then digested with papain extraction reagent for 3 h at 65 °C with 30 min interval mixing. The cell lysate from two wells was combined into one sample and transferred to a micro-centrifuge tube. Total 6 μ-masses were used as triplicated samples (2 μ-masses/ sample) for each experiment. Afterwards, the manufacturer’s protocol was followed.

### Quantitative Polymerase Chain Reaction (qPCR)

Total RNA was extracted from 10 to 12 μ-masses using TRIzol reagent (GIBCO-BRL/ Invitrogen by Life Technologies, Grand Island, NY, USA). Reverse transcriptase-polymerase chain reaction (RT-PCR) was performed with a SuperScript II reverse transcriptase (Invitrogen by Life Technologies, Grand Island, NY, USA) and random primer mix (NEB, Ipswich, MA, USA). For qPCR, C1000 thermal cycler system with CFX96 real-time PCR detection systems (Bio-Rad, Hercules, CA, USA) and IQTM SYPR® Green supermix (Bio-Rad, Hercules, CA, USA) were used with the following primers: type II collagen (forward, 5'-CCCTGAGTGGAAGAGTGGAG-3', reverse, 5'-GAGGCGTGAGGTCTTCTGTG-3'), type I collagen (forward, 5'-CGATGGCTGCACGAGTCACAC-3', reverse, 5'-CAGGTTGGGATGGAGGGAGTTTAC-3'), GAPDH (forward, 5'- TCGACAGTCAGCCGCATCTTCTTT-3', reverse, 5'-ACCAAATCCGTTGACTCCGACCTT-3'). The relative mRNA levels were evaluated using the 2^ΔΔC(t)^ method.

### Analysis of chondrocyte attachment to rabbit cartilage

The distal end of the femurs were removed and partial osteochondral defects (3 mm × 6 mm, 1 mm deep) were created in the trochlear groove of the femurs of adult rabbits. The cartilages with defects were cultured in serum-free DMEM (Gibco by Life Technologies, Grand Island, NY, USA) supplemented with antibiotic-antimycotic (Gibco by Life Technologies, Grand Island, NY, USA) for 24 h. To determine the length of time for cell attachment to the defect area, the cartilage defect on the femoral condyle was faced upward. CM-DiI (Molecular probes, by Life Technologies, Grand Island, NY, USA) labeled hChonJ and CM-DiO (Molecular probes, by Life Technologies, Grand Island, NY, USA) labeled irradiated-hChonJb#7 were used for the transplantation. The defect was filled with TG-C (mixed hChonJ:hChonJb#7, ratio = 3:1, 2 × 10^6^ cells in 10 μL PBS), and was left undisturbed for 7, 15, 20, 30, and 40 min. The femurs were then turned with the defect side down for 10 min in a 50 ml conical tube containing PBS to washout the non-adherent cells in the culture medium. Non-adherent cells in the medium were collected and cell number was counted. The number of cells attached to the cartilage defects was calculated by subtracting the non-adherent cell number from the initial cell number of 2 × 10^6^. The cells attached to the cartilage were observed with a fluorescent image analyzer LAS-4000 (FujiFilm, Tokyo, Japan). The use of blue LED with an Y515Di filter and red LED with a R670 filter allows the direct detection of fluorescence from CM-DiO labeled hChonJb#7 and CM-DiI labeled hChonJ, respectively [24]. Guidelines from the Institutional Animal Care and Use Committee at the Inha University Medical School and Hospital were followed during all animal procedures.

## Results

### hChonJb#7 cells produce TGF-β1 as functional as rhTGF-β1

Cysteine 223 and Cysteine 225 of TGF-β1preprotein are essential for dimerization and mutation of them to Serine was known to enhance spontaneous maturation without activation [25]. Based on this observation, we manipulated human TGF-β1 gene to bear the mutation and isolated single clone (hChonJb#7) after transduction into human chondrocyte (hChonJ). While we analyzed the DNA sequence of the transduced gene in hChonJb#7, we detected additional mutations with which the wild-type threonine at position 84 and arginine residue at position 255 of the TGF-β1 precursor had changed into alanine and tryptophan respectively (A84/W255) in addition to the intended mutation of Cysteine 223 and 225 into Serine (S223/255). To determine the effects of the mutations, we prepared pMSCV constructs bearing 4 different TGF-β1variants -wild type (WT), S223/225 mutant, A84/W255 mutant, and hChonJb#7 mutant (both S223/225 and A84/W255 mutated and designated as #7 in figures) - and transfected them into Cos-1 monkey kidney cells to obtain TGF-β1 preproteins released into its culture supernatant.

**Fig. 1:**
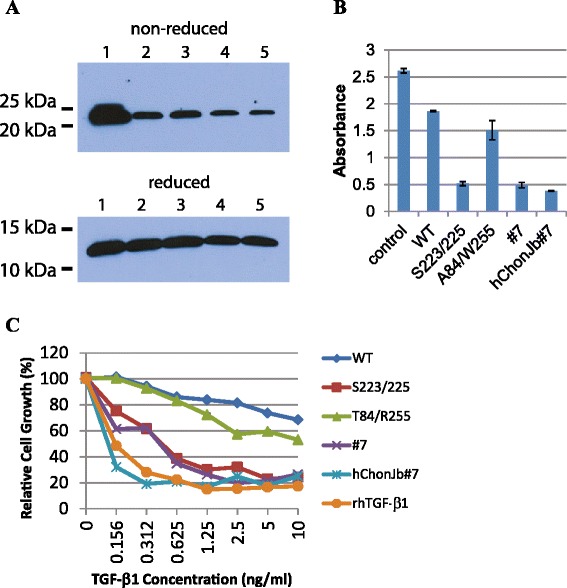
TGF-β1 produced from hChonJb#7 functions as rhTGF-β1. **a** TGF-β1gene was modified to bear WT or mutants related to hChonJb#7. Its expression was tested by western blot using culture medium of Cos-1 cells that were transfected with individual constructs. 1: rhTGF-β1, 2: pMSCV TGF-β1 (WT), 3: pMSCV TGF-β1 (S223/225 mutant), 4: pMSCV TGF-β1 (A84/W255 mutant), 5: pMSCV TGF-β1 (S223/225 & A84/W255 mutant, designated #7). Upper panel is showing matured dimer of TGF-β1in a non-reducing gel that was shifted into monomer form in a reducing gel (lower panel). **b** TGF-β1gene transfected Cos-1 cell culture medium or hChonJb#7 cell culture medium was used as TGF-β1source to inhibit proliferation of Hep G2 carcinoma cells in 5 ng/ml concentration. Control; no addition of any TGF-β1, WT; wild type TGF-β1, S223/225; Serine 223 and Serin225 changed mutant TGF-β1, A84/W255; Threonine 84 and Arginine 255 changed mutant TGF-β1, #7; combined mutant of S223/225 and A84/W255, hChonJb#7; TGF-β1 contained in hChonJb#7 culture medium. The absorbance of the Y-axis increases in proportion to the number of live cells. The error bars represent the standard deviation. **c** The serially diluted supernatant containing TGF-β1 in concentration of 10 ng/ml to 0.156 ng/ml was applied into Hep G2 cell culture to determine IC50 of each TGF-β1 variant

To confirm their expression, TGF-β1 preproteins in culture supernatants were revealed by SDS-PAGE in both non-reducing and reducing conditions. In non-reducing conditions in which disulfide bonds can be maintained, all four variants showed the mature dimer form of TGF-β1, which is comparable to recombinant human TGF-β1 (rhTGF-β1), and the mature dimer was shifted into monomer form under reducing conditions (Fig. 1a).

To distinguish the activities of TGF-β1 variants quantitatively, we monitored proliferation of hepatocellular carcinoma HepG2 cells when treated with individual variant. This assay was based on the fact that active TGF-β1 inhibits cell proliferation. As shown in Fig. 1b, S223/225 mutant and #7 mutant of TGF-β1 preprotein inhibited proliferation of Hep G2 cells more efficiently (at least 3 times more) than WT or A84/W255 mutant suggesting that S223/225 or #7 mutant was more efficiently activated than WT or A84/W255. TGF-β1 preprotein produced from hChonJb#7 cells also showed similar inhibitory effect on Hep G2 proliferation comparable to that of S223/225 or #7 (Fig. 1b).

In serial dilution studies to determine IC_50_, TGF-β1 preprotein in hChonJb#7 cell culture supernatant showed almost identical functional activity (<0.156 ng/ml of IC_50_) with rhTGF-β1, which exists in a fully active form (Fig. 1c). Based on these observations, we confirmed that hChonJb#7 cells produce almost fully active TGF-β1.

### TGF-β1 produced by hChonJb#7 functions on hChonJ as effective as rhTGF-β1

In previous experiments, we showed that TGF-β1 produced from hChonJb#7 cells is equally functional as rhTGF-β1 in inhibiting carcinoma cell proliferation. Next, we examined whether TGF-β1 from hChonJb#7 cells is similarly effective on human chondrocytes (hChonJ cells).

**Fig. 2:**
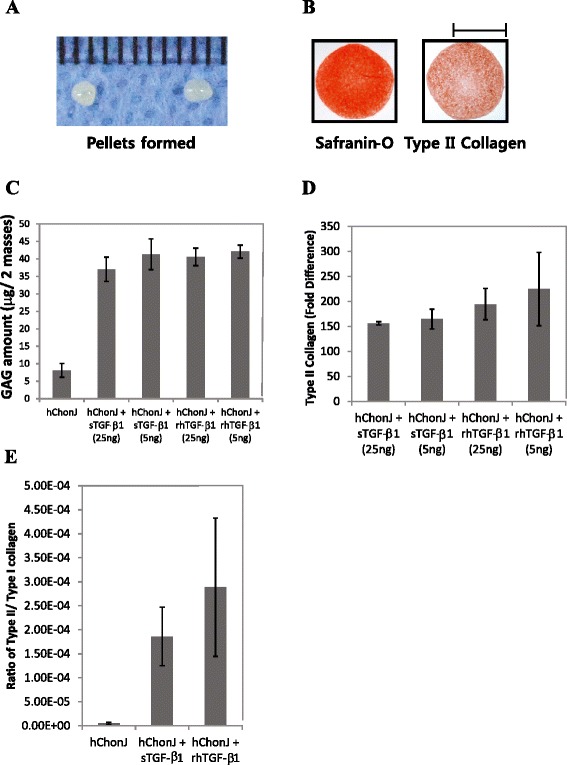
TGF-β1 produced from hChonJb#7 functions on hChonJ as much as rhTGF-β1. **a** hChonJ were treated with rhTGF-β1 in a 15 ml conical tube. After 3 weeks of incubation, pellets were formed in shiny and round shape (Grids: 1 mm interval). **b** The presence of proteoglycan in articular cartilage-like tissues of pellets were shown using S-O staining. The existence of type II collagen was confirmed with immunohistochemistry. The scale bar stands for 1 mm. **c** Different concentrations (5 ng or 25 ng/ mass) of rhTGF-β1 protein or the culture supernatant of hChonJb#7 which contains identical amounts of TGF-β1 was added while setting up the hChonJ chondrocyte micro-mass culture. After 1 week, total soluble GAG and sulfated proteoglycans were labeled with 1, 9-dimethylene blue and the content was measured at 650 nm wavelength in a micro-plate reader. The Y-axis shows GAG amount formed in two micro-masses. The error bars represent the standard deviation from three time repeated experiments. **d** The total RNA was isolated from the hChonJ micro-masses and used for qPCR to measure the expression level of type II collagen. The level of type II collagen was normalized against glyceraldehede-3-phosphate dehydrogenase (GAPDH) in each condition. The Y-axis shows fold difference of type II collagen mRNA level increased inTGF-β1 supplemented micro-masses compared to hChonJ micro-masses. The error bars represent the standard deviation from three time repeated experiments. **e** The Y-axis shows the increased ratio of type II collagen against type I collagen expression levels. The error bars represent the standard deviation from three time repeated experiments

When hChonJ cells were incubated with rhTGF-β1 in a pellet culture, a shiny white cartilage-like pellet was formed in 2 weeks (Fig. 2a). However, if not supplemented with rhTGF-β1 then pellets do not form well (data not shown). Safranin-O staining showed the presence of proteoglycan in articular cartilage-like pellets. Type II collagen was also detected using immunohistochemistry (Fig. 2b). Proteoglycan and type II collagen are the main components of extra-cellular matrix (ECM) abundant in articular cartilage so they are considered as markers for articular chondrocytes. Rather than staining or using immunohistochemistry, proteoglycan and type II collagen were detected quantitatively with alternative assays such as the GAG assay and qPCR, respectively, following micro-mass culture which is more convenient culture method than pellet culture. As shown in Fig. 2, rhTGF-β1 supplemented hChonJ micro-masses formed approximately 4 times more GAG compared to untreated masses. The TGF-β1 produced by hChonJb#7 exhibited a tendency to induce GAG formation at similar bioactivity levels compared to fully active rhTGF-β1 protein (Fig. 2c). There was a trend in the results of qPCR experiments to detect type II collagen mRNA levels which indicated that TGF-β1 produced from hChonJb#7 cells and rhTGF-β1 both induced type II collagen gene expression and did so at a similar scale exhibiting approximately a 200 fold difference compared to the negative control (Fig. 2d). Although the type II collagen expression level increased, if this was accompanied by an increase in type I collagen expression, it would be difficult to consider the chondrocytes as redifferentiated. Therefore, we examined the ratio of type II collagen mRNA levels over to type I collagen mRNA levels. As shown in Fig. 2e, the ratio also increased compared to the negative control suggesting that type II collagen gene expression was selectively induced.

### The main function of hChonJb#7 as the source of active TGF-β1

**Fig. 3:**
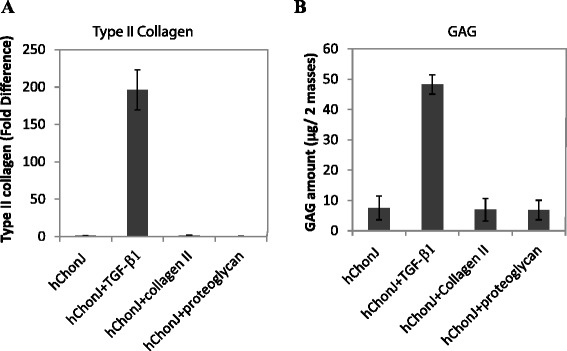
The main function of hChonJb#7 as the source of active TGF-β1. **a** The hChonJ micro-masses were cultured with medium including rhTGF-β1 (25 ng/ μ-mass), purified human type II collagen (25 ng/ μ-mass) or proteoglycan human joint cartilage extract (25 ng/ μ-mass). After 1 week, total RNA was purified from each micro-mass and applied to qPCR to measure the type II collagen gene expression level. The Y-axis shows fold difference of type II collagen mRNA level increased inTGF-β1 supplemented micro-masses compared to hChonJ micro-masses. The error bars represent the standard deviation. **b** Micro-masses cultured in the same method to (**a**) used to measure the accumulated GAG amount. The error bars represent the standard deviation

Because the hChonJb#7 cells themselves produce type II collagen [19], we tested whether the extra-cellular matrix of hChonJb#7 including type II collagen and proteoglycan could affect the phenotypic properties of hChonJ cells when they were mixed. To test this possibility, we added human type II collagen or proteoglycan into micro-mass culture as media supplements and compared this to rhTGF-β1. Unlike rhTGF-β1, neither type II collagen nor proteoglycan induced type II collagen gene expression (Fig. 3a). Next, we examined GAG accumulation using the same factors used to test for type II collagen change. hChonJ supplemented with rhTGF-β1 accumulated more GAG than the hChonJ control. However, neither type II collagen nor proteoglycan supplemented hChonJ micro-masses showed any additional GAG accumulation as compared to hChonJ only control (Fig. 3b). From these observations, we could assume the main function of hChonJb#7 in cartilage regeneration is as the source of active TGF-β1 to hChonJ cells.

### Type II collagen and GAG production in TG-C

**Fig. 4:**
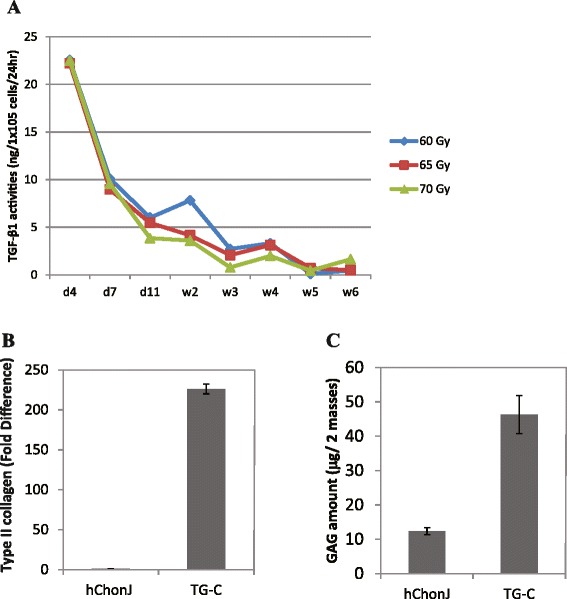
Type II collagen and GAG production by TG-C. **a** Expression level of TGF-β1 in irradiated hChonJb#7 cells was monitored for 6 weeks after the irradiation. **b** Type II collagen transcription level in hChonJ or TG-C micro-masses was determined by qPCR. Transcription level of type II collagen was normalized against GAPDH in each condition. The Y-axis shows the standardized fold difference between hChonJ and TG-C. The error bars represent the standard deviation. **c** GAG amount accumulated in hChonJ or TG-C micro-masses. TG-C masses accumulated about 4 times more GAG than hChonJ masses at the week 1 time point. The error bars represent the standard deviation

hChonJb#7 is genetically modified cell line produced via retro-viral infection and recombination. Previous uses of retroviral vectors in the clinic resulted in cases in which the patients who had been treated with retroviral gene therapy developed tumors, and most of these cases were due to the transduction into oncogenes [26]. Although the modified TGF-β1 gene was not introduced into any known oncogene in hChonJb#7, we decided to render hChonJb#7 cells replication-incompetent as an extra precaution. For this purpose, the hChonJb#7 cells were irradiated with weak dosage of X-ray irradiation so that the cell would die after completing its life span. However, it was expected to provide TGF-β1while it was alive. To test this, we measured the quantity of TGF-β1 produced by the hChonJb#7 cells after irradiation. As shown in Fig. 4a, a considerable amount of TGF-β1 was detected for approximately 2 weeks while the cells remained viable, confirming that the role of hChonJb#7 cells as TGF-β1 provider to hChonJ cells was not impaired.

To prove that hChonJ cells can be redifferentiated by irradiated hChonJb#7 cells, we mixed hChonJ cells and irradiated hChonJb#7 cells in 3:1 ratio (TG-C) and cultured them to form micro-masses. The 3:1 cell mixture ratio has been used for our pre-clinical animal study and Phase I and II clinical studies proving its efficacy and safety for cartilage regeneration [23, 27]. Together with rhTGF-β1, we measured type II collagen mRNA levels and the amount of GAG accumulated in the TG-C micro-masses to determine the hChonJ potency as articular chondrocytes in TG-C. The type II collagen mRNA level was approximately 200 fold higher (Fig. 4b) and the GAG amount was also at least 4 times more than negative control (Fig. 4c). These results were comparable to those of experiments performed with fully active rhTGF-β1 or hChonJb#7 culture supernatant (Fig. 2c and d) and we confirmed that irradiated hChonJb#7 cells also provided sufficient TGF-β1 to redifferentiate the hChonJ cells in TG-C.

### Adherence of chondrocytes to cartilage defects

**Fig. 5:**
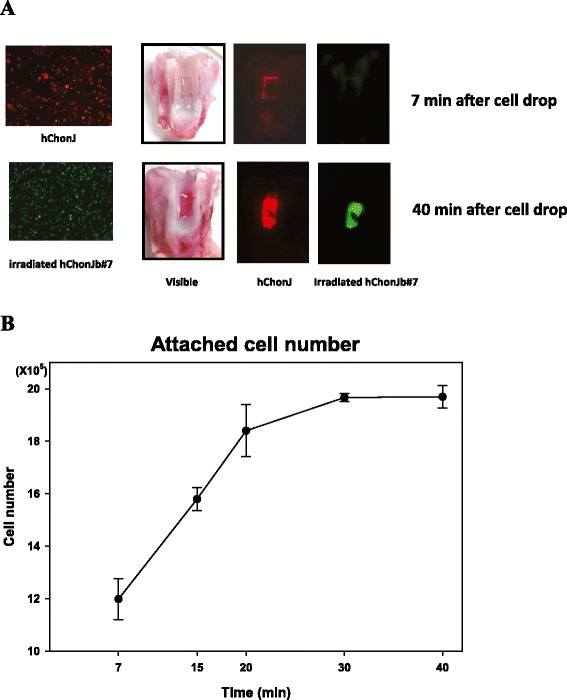
Attachment of chondrocytes to rabbit cartilage. **a** TG-C cells were detected in the cartilage which has an artificial defect on the surface. TG-C cells were labeled with fluorescent markers [CM-DiI (red color) labeled hChonJ and CM-DiO (green color) labeled irradiated hChonJb#7] and were analyzed with an image analyzer (LAS-4000, FujiFilm). **b** The number of cells attached to a cartilage defect was calculated by subtracting the non-adherent cells (collected from the culture media) from the initial number 2 × 10^6^

To measure the binding ability of TG-C to cartilage, fluorescent labeled hChonJ and hChonJb#7 cells were placed on cartilage with partial knee defects (rabbits). Both CM-DiI labeled hChonJ (red color) and CM-DiO labeled hChonJb#7 (green color) were bound to the defect area compared to the un-disturbed smooth area of the cartilage (Fig. 5a). The number of bound cells increased with time until it reached a plateau after 30 min (Fig. 5b). We also performed the same experiment in human knee joint tissue which was collected after knee joint replacement and observed the similar results (Data not shown). Based on these observations, we conclude that when TG-C is injected onto the damaged cartilage, irradiated hChonJb#7 and hChonJ cells attached to the lesion within 30 min and can play their role as a source of TGF-β1 and cartilage material in order to rebuild cartilage.

## Discussion

TGF-β1 was reported to stimulate chondrogenesis and synthesis of proteoglycan [28, 29]. However, the durability of articular cartilage-like material for full-thickness cartilage repair was not prolonged [30]. We speculated that this short durability was due to the one-time supply of TGF-β1. To provide TGF-β1 for a longer period, the TGF-β1 gene was modified to be activated spontaneously and introduced into chondrocytes by a retroviral vector. The transduced chondrocytes, hChonJb#7, were mixed with normal chondrocytes, hChonJ, in a ratio of 1:3 as TG-C to provide a treatment of the damaged cartilage. Recently, we have successfully completed a Phase II clinical study using TG-C. Preliminary data (not shown) from the Phase II study have demonstrated the efficacy of TG-C in regenerating cartilage as well as in relieving the pain and improving joint function in osteoarthritis patients.

In this study, to elucidate the mechanism of cartilage regeneration, the effect of TGF-β1 on type II collagen and GAG expression in micro-mass cultures without a scaffold was investigated. When the expressed TGF-β1 variants were tested for their cell proliferation inhibitory activity, modified TGF-β1 preprotein showed more efficient inhibition than wild type TGF-β1 preprotein (Fig. 1). The modified TGF-β1 produced from hChonJb#7 cells had activity comparable to fully active rhTGF-β1, suggesting that modified TGF-β1 preprotein was more efficiently activated than WT preprotein.

To determine if the modified TGF-β1 produced from hChonJb#7 cells is also effective on hChonJ cell redifferentiation, we developed a micro-mass culture method for chondrocytes and monitored the type II collagen mRNA level and the amount of GAG, which are functional markers for articular cartilage regeneration. For induction of type II collagen gene expression and production of GAG, the modified TGF-β1 produced from hChonJb#7 was as effective as rhTGF-β1 (Fig. 2). When hChonJb#7 cells were mixed hChonJ chondrocytes in TG-C, the type II collagen mRNA and the GAG amount was higher than for hChonJ cells alone, approximately 200 fold and 4 fold higher, respectively. The hChonJb #7 cells themselves also produce type II collagen and proteoglycan. Therefore, we tested whether type II collagen and proteoglycan could have additive effect on hChonJ redifferentiation, revealing that there was no indication of hChonJ redifferentiation when type II collagen or proteoglycan was added individually to chondrocyte cultures. Although they had no effect in changing hChonJ internal characteristics, they might play a physical role in adherence of the chondrocytes to the cartilage lesion.

Previously, there have been inconsistent results on the effect of TGF-β1 on the production of type I or type II collagen. Down-regulation of type II collagen gene expression by TGF-β1 was reported in articular chondrocytes [31, 32]. To the contrary, the re-appearance of type II collagen with the treatment of TGF-β1 was also reported [33, 34]. There have also been controversial or contradictory studies about the clinical effects of TGF-β. While some groups reported positive effects of TGF-β on osteoarthritis (OA) treatment [35, 36], others showed evidence that increased TGF-β was involved in OA progression or osteophyte formation. In addition, treatment of murine knee joints with multiple injections of TGF-β was reported to induce synovial fibrosis [37]. These differences in reported results may be due to a variety of factors including differences in TGF-β1 concentration, differences in the stage of OA being treated, or different injection locations. Therefore, a patients’ particular condition may affect the type and severity of effects. Our results from this study showed an increase in type II collagen mRNA expression level induced by TGF-β1 with a corresponding increase in the ratio of type II collagen to type I collagen (Fig. 2d and e). We further examined type X collagen mRNA levels, and obtained a similar result with type I collagen (data not shown). Our results support the premise that TGF-β1 is more likely to induce shifting of chondrocytes from a dedifferentiated osteoarthritic status to a more articular chondrocyte-like status. However, to preclude possible side effects due to a prolonged supply of TGF-β1, we rendered the hChonJb#7 cells replication-incompetent so that they can provide TGF-β1 only for a limited period, enough to induce the redifferentiation of hChonJ.

It has been shown that primary or very early passages of chondrocytes have the potential to bind to exposed areas in a cartilage defect [38]. We confirmed here that later passages of chondrocytes could also bind to the damaged area of cartilage (Fig. 5). The binding results indicate that a sufficient number of chondrocytes can adhere to a cartilage defect to produce a matrix in a three dimensional structure. Because this experiment was performed *ex vivo* using the distal end of the femurs removed from the rabbits, we cannot conclusively state that the experiment fully mimics the *in vivo* situation. However, the positive observations of cartilage formation from our pre-clinical and clinical studies support the hypothesis that attachment of chondrocytes is a potential factor in the mechanism of action. This binding ability of TG-C is very important since the need of a scaffold to localize chondrocyte in the lesion can be potentially avoided. Therefore, the binding of the cells in the chondrocyte and irradiated TGF-β1-expressing cell mixture (TG-C) could provide a rationale for the direct-injection of TG-C into a knee-joint without any scaffold.

In addition to chondrocyte localization, the scaffold usage was also shown to help allogeneic chondrocytes to avoid an immunogenic response. While suspended allogeneic chondrocytes induced immune responses, allogeneic osteochondral plugs or tissue-engineered grafts using allogeneic chondrocytes tolerated immunogenic response [39]. Especially, allograft chondrocytes embedded in type II collagen gel showed repair of rabbit articular surfaces with no sign of immunogenic reactions [40]. Therefore we surmise, based on the data shown in this study, that the successful regeneration of cartilage with TG-C from our previous studies may be due to the matrix-like effect of the increased type II collagen and GAG produced by the TG-C mixture. These components could have acted as a scaffold to avoid immune response. However, further investigation into the potential shielding from immune response by a scaffold effect should be followed.

We are proposing a model of cartilage regeneration by the co-injection of normal chondrocytes and irradiated chondrocytes that express TGF-β1 (TG-C). First, when TG-C is introduced into the knee joint, TG-C binds to the area of defective cartilage like scaffold. Second, the attached hChonJ produce more type II collagen and GAG due to the influence of TGF-β1 secreted by the hChonJb#7 cells. Third, the progression of cartilage regeneration is being aided by compacting chondrocytes and type II collagen–GAG matrices.

## Conclusions

Our data suggest that TG-C, the mixture of human chondrocytes and irradiated TGF-β1 producing chondrocytes, can function as a scaffold substitute by continuously providing type II collagen and GAG around chondrocytes to the damaged cartilage.
